# Serum NGF, BDNF and IL-6 Levels in Postpartum Mothers As Predictors of Infant Development: The Influence of Affective Disorders

**DOI:** 10.1371/journal.pone.0094581

**Published:** 2014-04-14

**Authors:** Karen Amaral Tavares Pinheiro, Ricardo Tavares Pinheiro, Fábio Monteiro da Cunha Coelho, Ricardo Azevedo da Silva, Luciana Ávila Quevedo, Cristina Carvalhal Schwanz, Carolina David Wiener, Gisele Gus Manfro, Márcia Giovenardi, Aldo Bolten Lucion, Diogo Onofre de Souza, Luis Valmor Portela, Jean Pierre Oses

**Affiliations:** 1 Postgraduate Program in Health and Behavior, Universidade Católica de Pelotas, Pelotas, Rio Grande do Sul, Brazil; 2 Department of Psychiatry, Universidade Federal do Rio Grande do Sul, Porto Alegre, Rio Grande do Sul, Brazil; 3 Department of Physiological Sciences. Universidade Federal de Ciências da Sáude de Porto Alegre, Porto Alegre, Rio Grande do Sul, Brazil; 4 Department of Biochemistry, Universidade Federal do Rio Grande do Sul, Porto Alegre, Rio Grande do Sul, Brazil; Universidade do Estado do Rio de Janeiro, Brazil

## Abstract

**Background:**

Early adverse experiences are associated with increased risk of developing psychiatric disorders, although little is known about the neurobiological mediators involved. The mechanisms by which early environmental influences may mediate vulnerability in the development of offspring await further investigation. The present study correlated the NGF, BDNF, IL-6 and cortisol levels of mothers with postpartum affective disorders (PPAD) with infant development.

**Methods:**

A longitudinal study was performed with 152 pregnant women and their infants. Between 60 and 120 days after delivery, women were interviewed and provided biological samples for biochemical analysis, and the infants were examined for neurobiological-motor development.

**Results:**

Overall, the mothers' history of affective disorders, PPAD and anxiety disorder were associated with infant motor development. Using an adjusted linear regression analysis, PPAD (p = 0.049), maternal anxiety disorder (p = 0.043), NGF level (p = 0.034) and infant cortisol level (p = 0.013) were associated with infant motor development. Using a factorial analysis of primary components, two components were retained. The psychological factor was characterized by a positive loading of a history of affective disorder, PPAD and anxiety disorder. For the biological factor, infant cortisol adhered negatively with infant motor development, but NGF was positively associated. The psychological factor had a negative association, but the biological factor had a positive association with infant motor development.

**Conclusions:**

There are few studies that have focused on the relationship of biomarkers and infant neurodevelopment. Our study points that psychological and biological factors are associated with infant motor development, however the causal relationship between these factors is still to be defined.

## Introduction

Several studies have demonstrated the influence of the mother on infant neurobiological development [1]. When the mother fails to provide adequate incentives to offspring in the first months of life, the chance of a significant loss in neurobiological and psychological development increases [2]. Maternal deprivation may occur due to postpartum affective disorders (PPAD) [3]. Mothers with affective disorder spend less time positively interacting with their children and show more inconsistent and ineffective child management strategies than non-depressed parents [4].

Early adverse experiences in humans are associated with increased risk for the development of psychiatric disorders, although little is known of the neurobiological mediators of this effect [5], [6]. Neurotrophins such as nerve growth factor (NGF) and brain-derived neurotrophic factor (BDNF), which play a fundamental role in brain function and neuroprotection and are affected by stress, are good candidates for the transduction of the effects of adverse events to changes in brain function [7]–[9]. Interleukin (IL)-6 is a proinflammatory cytokine and an important messenger molecule in the immune system that also influences the brain and the neuroendocrine system [10].

Human data and animal studies have suggested that the relationship between the quality of early environment and emotional responding is mediated by maternal influences on brain development [11]–[13]. The mother's modulatory function on environmental input is essential for the facilitation and inhibition of experience-dependent maturation of the child's developing biological structures, particularly neurobiological structures [14]. This function influences motor development (MD) at birth and continues during the first 12 months [15]–[17]. A study by Abbott et al. of the relationship between aspects of the household environment and children's motor development that also addresses the contribution of multiple subsystems to the acquisition of motor skills in children suggests that an environment with greater support and more stimuli is associated with higher levels of MD in children [18]. Early experiences that result in the disruption of the mother-infant relationship have long-term influences on behavioral and endocrine responses to stress [19].

The mother also plays an important role in the regulation of stress responsiveness of the offspring [19], [20]. Although most research on hypothalamic-pituitary-adrenal axis (HPA) regulation during ontogeny has focused on intrinsic regulatory factors, it appears that extrinsic factors also play an important role in this regulation. In particular, maternal factors appear to disrupt the homeostasis of the infant's HPA axis [21], [22]. Experiments by Denenberg in the 1960s demonstrated that the “emotional state” of the mother affected the emotional state of the offspring [23]. The so-called “maternal mediation” hypothesis, first proposed in the ‘70 s [24], states that changes in maternal behavior underlie the effects of early manipulations in the offspring. This hypothesis has been confirmed, and a direct relationship between variations in the level of maternal care and the development of individual differences in the behavioral and neuroendocrine responses to stress of the offspring has been described [25], [26].

The molecular mechanisms by which early environmental influences alter the circuits that may mediate the development of offspring are very complex and await proper investigation [27], [28]. The goal of the present study was to correlate NGF, BDNF, IL-6 and cortisol levels in mothers with affective disorders during the postpartum period with infant neurobiological development.

## Methods

From July to September 2008, a longitudinal study of pregnant women and their subsequent infants (i.e., dyads) was performed. The women were recruited from the Brazilian National System of Public Health in Pelotas, a southern Brazilian city. The subjects underwent prenatal follow-up, and the inclusion criteria included the following specifications: more than 18 years old, living in the urban area, not taking antidepressants or mood regulators and capable of understanding and completing the neuropsychiatric questionnaires in the postpartum period. Between 60 to 90 days after delivery, the women were re-interviewed at home and provided blood for NGF, BDNF, and IL-6 determination and saliva for cortisol analyses. Cortisol was measured using salivary levels in both mothers and infants. In the 4th month of life, infants were evaluated for neurobiological - motor development. All women answered a confidential self-administered questionnaire that included questions about socioeconomic conditions (according to the Economic Classification for Brazil of the Brazilian Association of Population Survey Companies, in which the highest-income level is “A” and the lowest “E”), age, previous psychiatric treatment, use of psychoactive drugs in the past, previous suicide attempts, smoking habits, alcohol consumption, living with the father and the type of delivery.

PPADs (depression, hypomanic/manic and mixed episode) and anxiety disorders were assessed using the Mini International Neuropsychiatric Interview (MINI Portuguese - version 5.0 Plus) [29]. MINI is a clinical structured interview that is compatible with DSM-IV criteria. It establishes a dichotomic variable for the presence or absence of PPAD.

Stressful life events (SLEs) were assessed by means of six questions obtained from life events and coping scales [30]. The women were questioned in the postpartum period about the following issues during the gestational period: the death of someone in the family, grave sickness, a change of address, unemployment, disengagement and the risk of abortion.

The Alberta Infant Motor Scale (AIMS) was used to evaluate the infants' neurobiological -motor development [31]. The AIMS, which is an observational assessment scale, is designed to measure gross motor maturation in infants from birth through independent walking (18 months). Based on the literature, 58 items were generated and organized into four positions: prone, supine, sitting and standing. Each item describes three aspects of motor performance: weight-bearing, posture and antigravity movements. Each participant's raw score was obtained by adding the scores of each scale. The raw scores and the ages of the infants were displayed in a graph in order to identify the percentile of infant neurobiological - motor development. In this study, we used the AIMS score for analysis. Three experienced physiotherapists who were blinded to the outcomes applied the AIMS testing. A concordance analysis was performed at the beginning and in the middle of the measurements.

Peripheral venous blood samples (5 ml) were collected in anticoagulant-free tubes (vacutainer system) from all subjects between 08:00 and 10:00 a.m. The samples were immediately centrifuged at 5,000×g for 10 min, and the serum was stored at −80°C until analysis. Serum levels of NGF, BDNF and IL-6 were measured using a commercially available enzyme immunoassay kit. The amounts of BDNF, NGF and IL-6 were determined by measuring the absorbance at 450 nm with a SpectraMax M5 spectrophotometer. All samples and standards were measured in duplicate, and the coefficient of variation was less than 5%. The serum NGF, BDNF and IL-6 levels are expressed as ng/ml.

Saliva samples (1 ml) were collected in sterile microtubes from all subjects between 08:00 and 10:00 a.m. The samples were immediately centrifuged at 5,000×g for 10 min, and the supernatant was stored at −80°C until analysis. Salivary cortisol was measured in duplicate samples with a commercially available High Sensitivity Salivary Cortisol enzyme immunoassay kit. The amount of cortisol was determined by measuring the absorbance at 450 nm as described above. All samples and standards were measured in duplicate, and the coefficient of variation was less than 5%. The salivary cortisol levels are expressed as µg/dl.

Data was double-entered into an Epi Info 6.04d software application, and an internal consistency check was conducted. Statistical analyses were performed using the Stata Statistical Package, version 9. Descriptive statistics were used to report the socio-demographic information. Clinical characteristics of the sample used to assess infant neurobiological - motor development (i.e., the AIMS Score) were analyzed using one-way ANOVA. Bonferroni correction and correlations within the AIMS scores and biological markers were analyzed using a Spearman correlation. The Spearman correlation was used because the distributions of biomarkers did not fill the requirements for normality. The variables included in bivariate analyses were as follows: in the first level, social class, maternal age, type of delivery, infant sex, prematurity, birth weight, smoking, alcohol consumed in last year; in the second level, history of affective disorders, postpartum affective disorder (PPAD), type of PPAD episode, mother anxiety disorder, mother BDNF level, mother NGF level, mother IL-6 level, mother and infant cortisol levels. Linear regression of infant neurobiological - motor development (i.e., the AIMS Score) was used for all variables with a p-value≤0.2 when associated with exposure and outcome. We considered associations with a p-value≤0.05 to be statistically significant. Furthermore, to determine the grouping of the associated factors with infant motor development, we conducted an exploratory factorial analysis. The extraction method was Primary Component Analysis (PCA). The varimax rotation was used to facilitate the data interpretation, retaining independence of the factors. Only the variables that had statistical significance in the regression analysis were included. Factorial loadings greater than or equal to 0.3 were used to establish the factor to which each variable adhered. The Kaiser-Meyer-Olkin (KMO) test was conducted to verify sample adequacy in relation to the factorial analysis of primary components, which was adequate when greater than or equal to 0.5. In the same direction, Bartlett's test of sphericity was performed and was considered significant when less than 0.05.

Finally, to obtain a fuller comprehension of the results, we performed another linear regression analysis with the generated factor scores in PCA.

This work was approved by the Catholic University of Pelotas (UCPel) Ethics Committee 2008/30-2007/29 CONEP 44/2008-reg14253. The subjects provided written informed consent to their participation in this study. The infants were enrolled only if the mother or a legal guardian provided a written informed consent. The women with detected psychiatric disorders were referred to the Psychiatry Service at the UCPel. Infants with low motor development were referred to the UCPel physiotherapy clinic.

## Results

A total of 160 women and their offspring were identified in the defined period; 5% of the women in the study did not allow their children to be evaluated. The mean age of the women was 24.51±6.09 years, and most of them (55.3%) belonged to socio-economic class “C”. Most of the participants (82.8%) were living with the baby's father. During pregnancy, 28.3% of the women smoked at least 1 cigarette/day, and 34.9% had consumed alcohol within the last month. Exclusive breastfeeding, at least in the first month, was observed in 90.1%. During the postpartum evaluation, unipolar depression was measured in 4.6% of the women, mania or hypomania in 5.3% and mixed episodes in 5.3%. The overall prevalence of PPAD was 15.1%; of the women affected, 60.9% had a history of affective disorder during the gestational period. A smaller portion (8.7%) had more than one previous episode and had used medication in the past. Age, smoking, alcohol consumption, living with the father of the baby, social class, type of delivery, breastfeeding, prematurity, birth weight, history of affective disorders and stressful life events were not associated with infant motor development. PPAD and anxiety disorder were associated with infant motor development (Table 1). Regarding the biomarkers, only NGF was associated with infant motor development, while the cortisol levels of the infants showed only a trend of association. The mothers' BDNF, IL-6 and cortisol levels were not associated with infant MD (Table 2).

**Table 1:** Sample characteristics according to infant motor development (AIMS Score).

Characteristics	N (%)	AIMS SCORE	p-value
**Social class**			0.533
A and B	15 (9.8%)	19.30 (±3.74)	
C	84 (55.3%)	19.43 (±4.07)	
D and E	53 (34.9%)	19.94 (±3.66)	
**Maternal age**			0.425
Less than 24 years old	83 (54.6%)	19.50 (3.93)	
24 or more years old	69 (45.4%)	18.93 (4.33)	
**Type of delivery**			0.862
Normal	87 (57.2%)	19.35 (3.98)	
Caesarean	65 (42.8%)	19.22 (4.38)	
**Infant sex**			0.852
Male	71 (46.7%)	19.32 (3.99)	
Female	81 (53.3%)	19.18 (4.37)	
**Prematurity**			0.573
No	147 (96.7%)	19.89 (2.00)	
Yes	5 (3.3%)	16.63 (4.38)	
**Birth weight**			0.418
2,500 g or more	146 (96.1%)	19.44 (4.03)	
Less than 2,499 g	7 (3.9%)	20.71 (4.02)	
**Breastfeeding** (at least 1 month)			0.514
Yes	137 (90.1%)	19.58 (4.06)	
No	15 (9.9%)	18.93 (3.76)	
**Breastfeeding in 4^th^ month**			0.681
Yes	94 (61.8%)	19.59 (3.90)	
No	58 (38.2%)	19.41 (4.11)	
**Smoking**			0.525
Never	87 (57.2%)	19.25 (4.32)	
In the past, not during pregnancy	22 (14.5%)	20.15 (3.24)	
In pregnancy and now	43 (28.3%)	18.60 (4.04)	
**Alcohol consumed in last year**			0.702*
No	99 (65.1%)	19.28 (±4.23)	
Yes	53 (34.9%)	18.90 (±3.50)	
**History of affective disorder**			0.147
No	136 (89.5%)	19.33 (±4.22)	
Yes	16 (10.5%)	17.53 (±3.84)	
**Stressful life events in pregnancy**			0.254
2 or less	133 (87.5%)	19.40 (±4.01)	
3 or more	19 (12.5%)	18.17 (±4.90)	
**PPAD**			0.003
No	129 (84.9%)	19.67 (±4.09)	
Yes	23 (15.1%)	16.86 (±3.69)	
**Type of PPAD episode**			0.001*
None	129 (84.8%)	19.67 (±4.09)	
Unipolar depression	7 (4.6%)	18.42 (±2.22)	
Mania	8 (5.3%)	17.22 (±4.08)	
Mixed episode	8 (5.3%)	14.85 (±3.89)	
**Anxiety**			0.000
No	132 (86.8%)	19.71 (±3.93)	
Yes	20 (13.2%)	15.88 (±4.15)	

*ANOVA, Bonferroni.

**Table 2:** Correlation analysis of biomarkers and infant motor development (AIMS Score).

Biomarkers	Mean (SD)	Correlation coefficient (r)	p-value
BDNF level	2.39 (1.28)	0.042	0.669*
NGF level	2.43 (1.27)	0.231	0.010*
IL-6 level	2.06 (1.37)	0.040	0.663*
Maternal cortisol level	1.12 (0.96)	0.118	0.220*
Infant's cortisol level	1.32 (1.73)	−0.152	0.086*

*Spearman Correlation.

Correlation analyses between the biomarkers were conducted. IL-6 and NGF had a positive correlation (r = 0.575; p = 0.001), and NGF had a positive correlation with infant motor development (r = 0.207; p = 0.026). IL-6 did not have a direct correlation with infant motor development (r = 0.040; p = 0.663). Nevertheless, NGF had an almost significant trend of negative correlation with infant cortisol levels (r = −0.147; p = 0.054). Breastfeeding for at least one month had a positive correlation with NGF (r = 0.194; p = 0.037) but was not associated with infant motor development (p = 0.290) or with PPAD (p = 0.111).

In an adjusted linear regression analysis performed with the variables that remained in the model (p<0.2), a history of affective disorder (p = 0.049), PPAD (p = 0.049), mother anxiety disorder (p = 0.043), NGF levels (p = 0.034) and infant cortisol levels (p = 0.013) were associated with infant motor development (Table 3).

**Table 3:** Linear regression of infant neurobiological - motor development (AIMS Score) and: history of affective disorders, postpartum affective disorder (PPAD), maternal anxiety disorder, maternal NGF levels and infant cortisol levels.

	Adjusted Linear Regression Coefficient (95% CI)	p-value
History of affective disorder	−1.07 (−3.64 to 1.48)	0.147
PPAD *	−1.35 (−2.70 to −0.08)	0.049
Maternal anxiety disorder *	−2.89 (−5.69 to −0.91)	0.043
Maternal NGF level *	0.59 (0.46 to 1.13)	0.034
Infant cortisol level *	−0.31 (−0.55 to −0.06)	0.013

*Variables adjusted for each other and for history of affective disorder.

**Figure 1:**
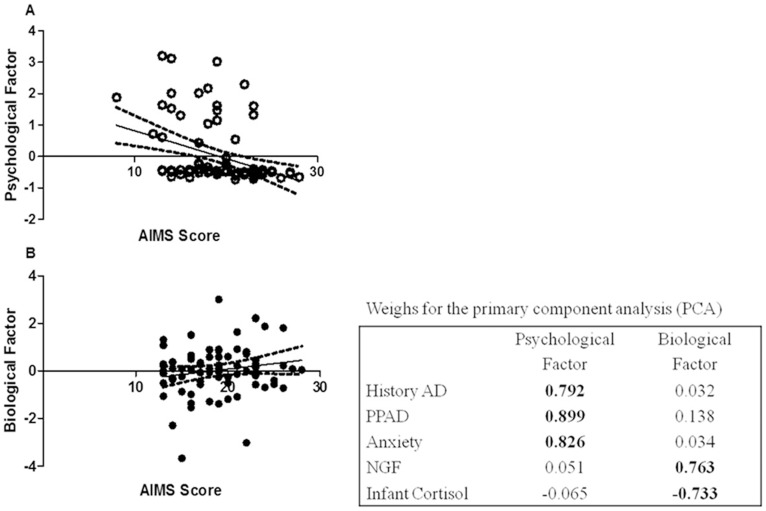
Primary component analysis (PCA) of psychological and biological factors in infants neurobiological-motor development according with Alberta Infant Motor Scale (AIMS). Panel A: Psychological factor including history of affective disorders (AD), postpartum affective disorders (PPAD), and anxiety influence in AIMS score. Panel B: Biological factor including maternal NGF levels and infant cortisol levels influence in AIMS score. The box shows the weight for each variable in psychological and biological component.

Figure 1 shows a primary component analysis (PCA). Bartlett's test was highly significant (p = 0.001) with a KMO of 0.63, indicating the validity of performing the analysis. In a factorial analysis of primary components, two components were retained. The explanation of the total data variance was 65.4%. Values with a load above 0.3 demonstrated with which factor each component adhered. We have designated Factor 1 as the psychological factor; this factor was characterized by the positive loading of a history of affective disorder, PPAD and anxiety disorder and explained 43.9% of the variance. In factor 2, which we call the biological factor, the components that adhered were infant cortisol level and NGF level; these explained 21.5% of the variance. Infant cortisol adhered negatively, but NGF adhered positively.

Table 4 shows the results of subjecting the score factors from the PCA to regression analysis. The psychological factor had a negative association, but the biological factor had a positive association with infant motor development.

**Table 4:** Linear regression of extract factor from Primary Component Analysis (PCA).

	Linear regression coefficient (95% CI)	p-value
Factor 1 – Psychological Factor	−1.486 (−2.25 to −0.72)	0.000
Factor 2 – Biological Factor	1.031 (0.35 to 1.70)	0.003

## Discussion

This study hypothesizes two distinct dimensions that represent the bases of infant neurobiological – motor development. The restrained factors – psychological and biological – explained 65.4% of the variance. This result is consistent with the results of other studies that have pointed to the importance of psychiatric illness in mothers and of biochemical influences on infant neurobiological development [1], [8]–[10]. Although this study is limited by the fact that the components observed here represent only a subset of the psychological and biological aspects involved in the process of infant motor development, the consistency of the findings of this study must be considered.

Similar to the findings of previous studies [4], the mothers in our sample with PPAD or anxiety disorder tended to have offspring with low motor development. In the original experiments by Denenberg and colleagues, it was clearly shown that the mother-infant relationship had decisive effects on infant development. In particular, the “emotional state” of the mother affected the offspring [23]. The mother's psychological sickness interferes with her ability to take care of the infant [32]. In other words, healthy mothers are more capable of becoming involved with their offspring and providing an appropriate emotional environment. The healthy mothers in our study had increased levels of NGF, and we observed an association with better infant motor development. These results may possibly be explained by the study about falling in love from Emanuele and collaborators [33]. According to their findings, NGF levels are significantly higher in subjects in love. This study suggests that elevated NGF levels may be related to specific emotions, such as intensely focused attention on a preferred individual, emotional dependency on and craving for emotional union with this beloved, and euphoria and increased energy to maintain the relationship. Our results could be hypothetically explained by considering that the mother-infant relationship is a love relationship and that when the mother is capable of falling in love with her offspring, this infant will have better development.

The importance of early affective interactions in development, although already described in the work of Freud and other pioneers of the study of development, has been given specific attention only in recent years [32], [34]. Although the theory of love has been little explored and is still incomplete [33], it is possible that certain aspects of the mother-infant relationship might be explained by changes in levels of oxytocin. This hormone has been called an attachment hormone because of its relationship to maternal bonding. Several studies have found that higher levels of this hormone are associated with better maternal bonding [35]–[36]. In our study, we did not analyze oxytocin levels. However, associations of oxytocin and NGF levels have been reported. Luppi has pointed out that the high levels of oxytocin present during labor and lactation can increase NGF levels in animal models [37].

The NGF levels of the subjects in this study contrast with data collected in other studies and suggest that different emotional states might be associated with changes in NGF levels [38]. In preclinical studies that model early adversity, maternal separation stress affects the levels of NGF, BDNF and cortisol in the limbic areas of offspring and produces long-lasting changes in emotional behavior and impaired responses to stress, suggesting that these neurotrophins may participate in the mechanism that underlies social bonding [39]–[43]. The fact that we did not collect blood samples from the infants is a limitation of our study.

We observed no association of maternal BDNF levels with PPAD or infant motor development or of maternal NGF levels with PPAD. This may be because the PPAD in the mothers of our sample was at an early stage. Kauer-Santana et al. found that neurotrophin levels were decreased only in the late stage of affective disorders [44]. Moreover, the methodological design of the present study may have prevented us from drawing conclusions regarding the role of neurotrophins and PPAD. The established relationship between increased levels of NGF and better scores for motor development suggests that mothers who were capable of responding to a stressful situation such as gestation, delivery and/or child-rearing had elevated NGF levels in response to a crisis and, as a result, were ready to get involved with their children. In our sample, maternal NGF levels were negatively correlated with infant cortisol levels; this observation corroborates the hypothesis of an adequate response of healthy mothers. In a study conducted in southern Brazil, Motta et al. demonstrated that the cortisol levels of infants whose mothers had affective disorder were significantly higher than those of controls; this shows that the functioning of the HPA axis is increased in the infants of mothers with affective disorder [45]. Carlson & Earls observed the motor development of infants between 2 and 9 months of age. They pointed out that, compared to infants who lived with parents, institutionalized children had poorer neuromotor development. The morning cortisol levels of all the children in their study showed an inverse correlation with motor and mental development as measured by the Bayley scales [46].

Few studies have focused on the relationship of neurotrophins and infant neurodevelopment [32]. Rather than a simple interface, this relationship is likely to be a complex one in which various aspects are correlated and influence each other. Until now, the genesis of this matter and how much one component influences another has been unknown.

Studies on the relationship between neurotrophins and their receptors and effects are being conducted, but a great deal of the information that has emerged from these studies remains to be interpreted. One must look beyond the specific cascade that is being activated [47]. The factors studied in the present work are markers, and use of these markers alone does not provide an adequate means of evaluating psychological illness and its repercussions on infants. Taken together, the biological and psychological components described in the present study provide a more realistic idea of how the complex process of infant development occurs, considering that individual neurobiological factors are only a single part of the developmental context.
